# Quality assessment of tandem mass spectra using support vector machine (SVM)

**DOI:** 10.1186/1471-2105-10-S1-S49

**Published:** 2009-01-30

**Authors:** An-Min Zou, Fang-Xiang Wu, Jia-Rui Ding, Guy G Poirier

**Affiliations:** 1 Department of Mechanical Engineering, University of Saskatchewan, 57 Campus Dr., Saskatoon, SK, S7N 59A, Canada; 2 Division of Biomedical Engineering, University of Saskatchewan, 57 Campus Dr., Saskatoon, SK, S7N 59A, Canada; 3 Health and Environment Unit, Laval University Medical Research Center (CHUL), Faculty of Medicine, 2705 Boul. Laurier, Quebec, QC, GIV 4G2, Canada

## Abstract

**Background:**

Tandem mass spectrometry has become particularly useful for the rapid identification and characterization of protein components of complex biological mixtures. Powerful database search methods have been developed for the peptide identification, such as SEQUEST and MASCOT, which are implemented by comparing the mass spectra obtained from unknown proteins or peptides with theoretically predicted spectra derived from protein databases. However, the majority of spectra generated from a mass spectrometry experiment are of too poor quality to be interpreted while some of spectra with high quality cannot be interpreted by one method but perhaps by others. Hence a filtering algorithm that removes those spectra with poor quality prior to the database search is appealing.

**Results:**

This paper proposes a support vector machine (SVM) based approach to assess the quality of tandem mass spectra. Each mass spectrum is mapping into the 16 proposed features to describe its quality. Based the results from SEQUEST, four SVM classifiers with the input of the 16 features are trained and tested on ISB data and TOV data, respectively. The superior performance of the proposed SVM classifiers is illustrated both by the comparison with the existing classifiers and by the validation in terms of MASCOT search results.

**Conclusion:**

The proposed method can be employed to effectively remove the poor quality spectra before the spectral searching, and also to find the more peptides or post-translational peptides from spectra with high quality using different search engines or *de novo *method.

## Background

With the development of proteomics, tandem mass spectrometry (MS/MS) has been used for the rapid identification and characterization of protein components of complex biological mixtures. Several database search programs such as SEQUEST [1] and MASCOT [2] have been developed to identify peptides by comparing the mass spectra obtained from unknown proteins or peptides with theoretically predicted spectra derived from protein databases. However, it is well known that these search programs produce a significant number of incorrect peptide assignments and leave the majority of spectra uninterpreted. One of the reasons this happens is that the majority of spectra generated from a mass spectrometry experiment are of too poor quality to be interpreted. The process of evaluating peptide assignments often relies on time-consuming and experience-dependent manual verification. Hence a filtering algorithm that removes those spectra with poor quality prior to the database search is appealing.

During the past few years, there have been a number of studies concerning the evaluation of the results of various search programs. Moore *et al*. described a probabilistic scoring scheme called Qscore to evaluate SEQUEST database search results [3]. Keller *et al*. applied the expectation maximization algorithm to estimate the accuracy of peptide identifications [4]. Anderson *et al*. employed the support vector machine (SVM) to distinguish between correctly and incorrectly identified peptides obtained by SEQUEST search program [5]. Razumovskaya *et al*. developed a method by combining a neural network and a statistical model to normalize SEQUEST scores and to provide reliability estimation for SEQUEST hits [6]. More recently, Nesvizhskii *et al*. described a dynamic quality scoring approach for finding high quality unassigned spectra in large shotgun proteomic datasets [7].

The earliest work concerned with the quality assessment of tandem mass spectra prior to database search was reported by Tabb *et al*. [8]. They assessed the spectral quality by use of some simple rules such as minimum and maximum thresholds on the number of peaks and a minimum threshold on total peak intensity. They claimed that such rules could remove 40% or more of the poor quality spectra. Purvine *et al*. used a pre-filtering algorithm named SPEQUAL with three features for tandem mass spectral quality assessment [9]. These three features were charge state differentiation, total signal intensity, and signal-to-noise estimates. They claimed that 55% of the poor quality spectra could be safely eliminated from further analysis by employing the SPEQUAL algorithm. Bern *et al*. proposed two different classification schemes for the automatic spectral quality assessment [10]. One scheme used the linear Fisher analysis to construct a classifier based on seven features including Npeaks, Total Intensity, Good-Diff Fraction, Isotopes, Complements, Water Losses, and Intensity Balance. The other one employed the SVM classifier based on observed mass/charge (*m*/*z*) ratios. The best result reported by Bern *et al*. [10] is that their SVM based classifier could remove 75% of the poor quality spectra while losing 10% of the high quality ones.

More recently, Flikka *et al*. [11] presented a filtering algorithm to eliminate the poor quality spectra before the database search. They tested and compared several classifiers on various proteome datasets (Q-TOF, ESI IT, and MALDI-TOF) from different instruments, and the best results from the classification test using ESI IT dataset showed that 83% of the poor quality spectra could be removed while losing 10% of the high quality ones. Salmi *et al*. [12] proposed a pre-filtering scheme for evaluating the quality of spectra before the database search, and they obtained the minimum false positive rate (FPR) of 25% while fixing the true positive rate (TPR) at 90%. Na *et al*. [13] proposed a machine learning approach to assess spectral quality by use of three spectral features which were Xrea based on cumulative intensity normalization and Good-Diff Fraction proposed by Bern *et al*. [10] for singly charged and doubly charged fragment ions. Na *et al*. [13] claimed that their method could filter out 75% of poor quality spectra while losing 10% of high quality ones when evaluating it on the ISB dataset. In [14], a probability based approach called msmsEval was proposed to assess the quality of tandem mass spectra. Using the ISB dataset as the classification test data, the TNR was obtained at about 83% while the TPR was 90%. This paper investigates the quality assessment of tandem mass spectra. The spectra are classified into two groups: high quality and poor quality spectra. In general, a spectrum is called to be of high quality if it is able to be identified by some methods, and otherwise it is called to be of poor quality. Several spectral features are proposed for the classification, and the SVM is applied to solve this classification problem. The results of computational experiments on two different mass spectral datasets (ISB and TOV) show that the proposed method can remove the majority of the poor quality spectra while losing a small minority of the high quality ones.

## Materials and methods

### Spectral features

A mass spectrum usually contains tens to hundreds of *m*/*z *values on the *x*-axis, each with corresponding signal intensity on the *y*-axis. In this study, after removing the noisy peaks by use of the morphological reconstruction method [15], 16 spectral features are introduced as follows for a spectrum.

*F*_1_: The number of peaks in the spectrum, square root-transformed.

*F*_2_: The average raw intensity of the peaks in the spectrum, log-transformed.

*F*_3_: The number of peaks with relative intensity >0.1, square root-transformed. In this study, the relative intensity of each peak is defined as the peak's intensity divided by the intensity of the highest peak.

*F*_4_: The average raw intensity of the peaks with relative intensity >0.1, log-transformed.

The log or square root transformation of the above spectral features was employed to obtain a more symmetric shape of the distribution and to minimize the variance across spectra in a mass spectral dataset. The experiments also verified that such transformation improved the performance of the spectral quality assessment by using the proposed SVM method.

To develop the remaining 12 features, four variables for a given peptide mass spectrum *S *are defined as

(1) *dif*_1_(*m*(*x*), *m*(*y*)) = *m*(*x*) - *m*(*y*)

(2) $$dif_{2}(m(x),m(y))=m(x)-\frac{m(y)+m(H)}{2}$$

(3) *sum*_1_(*m*(*x*), *m*(*y*)) = *m*(*x*) + *m*(*y*)

(4) $$sum_{2}(m(x),m(y))=m(x)+\frac{m(y)+m(H)}{2}$$

where *m*(*x*) and *m*(*y*) denote the *m*/*z*-values of peaks *x *and *y *in the spectrum *S*, respectively; *m*(*H*) is the mass of a hydrogen atom. A weighting factor is defined as

(5) $$W(x,y)=\frac{I_{r}(x)+I_{r}(y)}{2}$$

where *I*_*r*_(*x*) and *I*_*y*_(*x*) represent the relative intensities of peaks *x *and *y *in the spectrum *S*, respectively.

*F*_5 _- *F*_7_: Amino acid distances. These features measure how likely two peaks in a spectrum *S *differ by one of the twenty amino acids. Define

(6) *F*_5 _= ∑{*W*(*x*, *y*)|*di f*_1_(*m*(*x*), *m*(*y*)) ≈ *M*_*i*_, *i *= 1, 2,⋯,17}

(7) *F*_6 _= ∑{*W*(*x*, *y*)|*di f*_1_(*m*(*x*), *m*(*y*)) ≈ *M*_*i*_/2, *i *= 1, 2,⋯,17}

(8) *F*_7 _= ∑{*W*(*x*, *y*)|*di f*_2_(*m*(*x*), *m*(*y*)) ≈ *M*_*i*_/2, *i *= 1, 2,⋯,17}

where *M*_*i*_(*i *= 1, 2,⋯,17) are the 17 different masses of all 20 amino acids. This study considers all Methionine amino acids to be sulfoxidized and does not distinguish three pairs of amino acids in their masses: Isoleucine vs. Leucine, Glutamine vs. Lysine, and sulfoxidized Methionine vs. Phenylalanine since the masses of each pair are very close. The comparison implied by ≈ employs a tolerance, which was set to ± 0.5 Da for fragment ions and ± 2 Da for parent mass in this paper. The feature *F*_5 _measures the presence of peak pairs of singly charged ions corresponding to an amino acid mass difference in the spectrum *S*; the feature *F*_6 _measures the presence of peak pairs of doubly charged ions corresponding to an amino acid mass difference in the spectrum *S*, and the feature *F*_7 _measures the presence of peak pairs of one doubly charged and the other singly charged ions corresponding to an amino acid mass difference in the spectrum *S*. The use of the weighting factors in the features is to account the increased likelihood of more intense peaks being true fragment ions.

*F*_8 _- *F*_10_: Complements. These features measure how likely an N-terminus ion and a C-terminus ion in the spectrum *S *are produced as the peptide fragments at the same peptide bond. Define

(9) *F*_8 _= ∑{*W*(*x*, *y*)|*sum*_1_(*m*(*x*), *m*(*y*)) ≈ *M*_*p *_+ 2*m*(*H*)}

(10) *F*_9 _= ∑{*W*(*x*, *y*)|*sum*_1_(*m*(*x*), *m*(*y*)) ≈ *M*_*p*_/2 + 2*m*(*H*)}

(11) *F*_10 _= ∑{*W*(*x*, *y*)|*sum*_2_(*m*(*x*), *m*(*y*)) ≈ *M*_*p*_/2 + 2*m*(*H*)}

where *M*_*p *_is the mass of the precursor ion of the spectrum *S*. The feature *F*_8 _measures the presence of complementary peak pairs of singly charged ions in the spectrum *S*; the feature *F*_9 _measures the presence of complementary peak pairs of doubly charged ions in the spectrum *S*, and the feature *F*_10 _measures the presence of complementary peak pairs of one doubly charged and the other singly charged ions in the spectrum *S*.

*F*_11 _- *F*_13_: Water or ammonia losses. These features measure how likely one ion in the spectrum *S *is produced by losing a water or ammonia molecule from a b-ion or y-ion. Define

(12) *F*_11 _= ∑{*W*(*x*, *y*)|*di f*_1_(*m*(*x*), *m*(*y*)) ≈ *M*_*w *_or *M*_*a*_}

(13) *F*_12 _= ∑{*W*(*x*, *y*)|*di f*_1_(*m*(*x*), *m*(*y*)) ≈ *M*_*w*_/2 or *M*_*a*_/2}

(14) *F*_13 _= ∑{*W*(*x*, *y*)|*di f*_2_(*m*(*x*), *m*(*y*)) ≈ *M*_*w*_/2 or *M*_*a*_/2}

where *M*_*w *_and *M*_*a *_are the masses of a water molecule and an ammonia molecule, respectively. The feature *F*_11 _measures the presence of peak pairs of singly charged ions with a difference of a water or ammonia molecule in the spectrum *S*; the feature *F*_12 _measures the presence of peak pairs of doubly charged ions with a difference of a water or ammonia molecule in the spectrum *S*, and the feature *F*_13 _measures the presence of peak pairs of one doubly charged and the other singly charged ions with a difference of a water or ammonia molecule in the spectrum *S*.

*F*_14 _- *F*_16_: Supportive ions. These features measure how likely one ion in the spectrum *S *is a supportive ion. This paper considers two kinds of supportive ions a-ions and z-ions. Define

(15) *F*_14 _= ∑{*W*(*x*, *y*)|*di f*_1_(*m*(*x*), *m*(*y*)) ≈ *M*_*CO *_or *M*_*NH*_}

(16) *F*_15 _= ∑{*W*(*x*, *y*)|*di f*_1_(*m*(*x*), *m*(*y*)) ≈ *M*_*CO*_/2 or *M*_*NH*_/2}

(17) *F*_16 _= ∑{*W*(*x*, *y*)|*di f*_2_(*m*(*x*), *m*(*y*)) ≈ *M*_*CO*_/2 or *M*_*NH*_/2}

where *M*_*CO *_and *M*_*NH *_are the masses of a CO group and an NH group, respectively. The feature *F*_14 _measures the presence of peak pairs of singly charged ions with a difference of a CO or NH group in the spectrum *S*; the feature *F*_15 _measures the presence of peak pairs of doubly charged ions with a difference of a CO or NH group in the spectrum *S*, and the feature *F*_16 _measures the presence of peak pairs of one doubly charged and the other singly charged ions with a difference of a CO or NH group in the spectrum *S*.

The four features *F*_*i*_(*i *= 5, 8, 11, 14) represent the evidence of the existence of singly charged ions, and the eight features *F*_*i*+1 _and *F*_*i*+2_(*i *= 5, 8, 11, 14) represent the evidence of the existence of doubly charged ions.

These twelve features are developed according to the properties of the theoretical spectra proposed in our previous study [16] where the peak intensities have not been considered though. The experiments in this study showed that the use of the peak intensities improved the performance of the spectral quality assessment by using the SVM method. In general, the high quality spectra are expected to have larger values of these twelve features than those of the poor quality spectra. In addition, the more intense the peak pairs, the larger the values of these twelve spectral features are. At this point, 16 spectral features are introduced to describe the spectral quality. It is noted that the larger the number of the spectral peaks, the larger the values of the spectral features *F*_3 _and *F*_5 _- *F*_16 _are. This likely leads to a low sensitivity of the classifier as the high quality spectra for a spectrum with smaller number of peaks that would have smaller values of spectral features *F*_3 _and *F*_5 _- *F*_16_. To alleviate these effects, these spectral features are transformed as

(18) $$\frac{\log(1+F_{i})}{\delta +F_{1}},i=3,5,6,\cdots ,16$$

where *δ *is a small positive constant, and is set *δ *= 0.01 in this study. In a spectrum, a possible *m*/*z *range in which doubly charged ion peaks exist is less than a half of its peptide mass. Therefore, while we compute features *F*_6_, *F*_12_, and *F*_15_, the following conditions should be satisfied

(19) $$m(x)<\frac{M_{p}+m(H)}{2}\text{ and }m(y)<\frac{M_{p}+m(H)}{2}$$

Classification method

In this paper, the support vector machine is applied to assess the spectral quality because of its good generalization ability. The SVM was proposed by Vapnik based on the statistical learning theory [17]. An important characteristic of the SVM is that "while most classical neural network algorithms require an ad hoc choice of system's generalization ability, the SVM approach proposes a learning algorithm to control the generalization ability of the system automatically" [18]. The training of an SVM requires the solution of a quadric programming (QP) optimization problem, which is a large-scale system optimization problem. The sequential minimal optimization (SMO) decomposes the overall QP problem into fixed-size QP sub-problems (each involves only two Lagrange multipliers), and these sub-problems are solved analytically [19]. The SMO algorithm is one of the efficient algorithms for solving the large QP problem, which is employed to train the SVM in this work.

For an input vector *x *∈ *R*^*n *^(*n *= 16 in this paper), a decision can be made by a well-trained SVM as

(20) $$f(x)=\text{sign}\left(\sum_{i=1}^{l}\alpha_{i}y_{i}K(x_{i},x)+b\right)\equiv \text{sign}(g(x))$$

where *x*_*i *_∈ *R*^*n*^(*i *= 1, 2,⋯, *l*) are the support vectors; *α*_*i*_(*i *= 1, 2,⋯, *l*)) are the Lagrange multipliers; *y*_*i *_∈ {-1, +1}(*i *= 1, 2,⋯, *l*)) are the corresponding class of pattern for the support vector *x*_*i*_, and -1 for poor quality spectra and +1 for high quality spectra in this paper; *K*(*x*_*i*_, *x*)(*i *= 1, 2,⋯, *l*)) are the kernel functions, and *b *is the threshold. In this paper, the non-thresholded output *g*(*x*) is employed to generate the receiver operating characteristics (ROC) curve by using the algorithm proposed by Fawcett [20]. To evaluate the performance of the SVM classifiers, two correct rates are calculated in this study: true positive rate (TPR) and true negative rate (TNR)

(21) $$\text{TPR}=\frac{\text{TP}}{\text{TP}+\text{FN}}$$

(22) $$\text{TNR}=\frac{\text{TN}}{\text{TN}+\text{FP}}$$

where TP is the number of true positives; FP is the number of false positives; TN is the number of true negatives, and FN is the number of false negatives.

### Experimental data

This study used two different proteome datasets: the ISB dataset and the TOV dataset.

#### ISB dataset

The ISB dataset used in this study was acquired on an ion trap and was provided by the Institute of Systems Biology (ISB, Seattle, USA). The ISB dataset consists of 37043 peptide collision-induced dissociation (CID) mass spectra, which were generated by the tryptic digestion of a control mixture of standard 18 proteins (not of human origin) [4]. These spectra were searched against a human protein database (extracted from [21]) appended with the sequences of the 18 standard proteins and other common contaminants (totally, 5395 protein sequences in the final database) using SEQUEST search program. The single charged spectra were excluded from this study as the number of the singly charged spectra (only 504 singly charged) is too small. The distribution of multiple charged spectra is shown in Table 1. 'H' represents the number of the high quality spectra, and 'P' represents the number of the poor quality spectra. For the ISB dataset, this study has trained three SVM classifiers, one for doubly charged, one for triply charged, and the other for multiply charged spectra (not distinguishing between the doubly charged and triply charged ions).

**Table 1:** The distribution of multiply charged spectra in the ISB dataset

	H*	P*	Total
Doubly charged	1242	17253	18495

Triply charged	573	17471	18044

Total	1815	34724	36529

*A doubly charged spectrum is of high quality if its SQUEST Xcorr score is greater than 2.5, and otherwise it is of poor quality. A triply charged spectrum is of high quality if its SQUEST Xcorr score is greater than 3.5, and otherwise it is of poor quality.

#### TOV dataset

This dataset consists of 13467 peptide CID mass spectra which were acquired on an LCQ DECA XP ion trap (Thermo Electron Corp.) in Eastern Quebec Proteomic Center in Laval University Medical Research Center in Canada. The samples analyzed were generated by the tryptic digestion of a whole-cell lysate from the human malignant epithelial ovarian tumor cell-line TOV-112D [22]. These spectra were searched against a subset of the Uniref100 database, release 1.2 [23] including 44278 human protein sequences using SEQUEST. The assignments of 866 spectra were verified to be correct by PeptideProphet with the cut-off score of 0.8 [4], and were labeled as "high" quality spectra. The other 12601 spectra in TOV were labeled as poor quality. The distribution of these is shown in Table 2. For the TOV dataset, this study has only trained a classifier for the doubly charged spectra because the number of the high quality singly charged and triply charged spectra is too small to train a reasonable classifier.

**Table 2:** The distribution of spectra in the TOV dataset

	H*	P*	Total
Singly charged	10	917	927

Doubly charged	667	5575	6242

Triply charged	189	6109	6298

Total	866	12601	13467

*A spectrum is of high quality if its PeptideProphet score is greater than 0.8, and otherwise it is of poor quality.

## Results and discussion

Four separate SVM classifiers were trained in this study. The first SVM was trained for the doubly charged spectra; the second one was trained for the triply charged spectra; the third one was trained for the multiply (both doubly and triply) charged spectra. These three classifiers were trained based on the ISB dataset. The fourth one was trained for the doubly charged spectra of the TOV dataset. This study employed the radial basis functions (RBF) whose width parameter was set equal to 0.1 as the kernel functions of these four SVMs. The penalty term for the training set errors was set to 100.

It is noted that the class distribution of the tandem mass spectra is highly imbalanced, i.e., the number of the poor quality spectra is much larger than that of the high quality spectra. If one randomly chosen a certain number of spectra from the dataset to train an SVM, one would obtain a higher TNR and a lower TPR by the trained SVM classifier. To get a higher TPR, a larger number of high quality spectra are required to train an SVM classifier, which results in a significant increase of the number of the training samples. Therefore, much longer time will be taken to train the SVM. To overcome these problems and give an equal opportunity to the high quality and the poor quality spectra for the training of the SVM, this study employed the same number of high quality and poor quality spectra as the training samples. For example, in the ISB dataset only 860 (430 high quality and 430 poor quality) spectra were used to train the SVM classifiers for the doubly charged spectra while 600 (300 high quality and 300 poor quality) spectra were used to train the SVM classifier for triply charged spectra. The number of the samples in the training and test sets for all SVM classifiers is shown in Table 3. 'SVM2ISB' stands for the SVM classifier for the doubly charged ISB spectra; 'SVM3ISB' stands for the SVM classifier for the triply charged ISB spectra; 'SVMMISB' stands for the SVM classifier for the multiply charged ISB spectra, and 'SVM2TOV' stands for the SVM classifier for the doubly charged TOV spectra.

**Table 3:** The number of the samples in the training and test sets

SVM classifier	Training set (H:P)	Test set (H:P)
SVM2ISB	430:430	812:16833

SVM3ISB	300:300	273:17171

SVMMISB	605:605	1210:34623

SVM2TOV	350:350	317:5225

**Figure 1:**
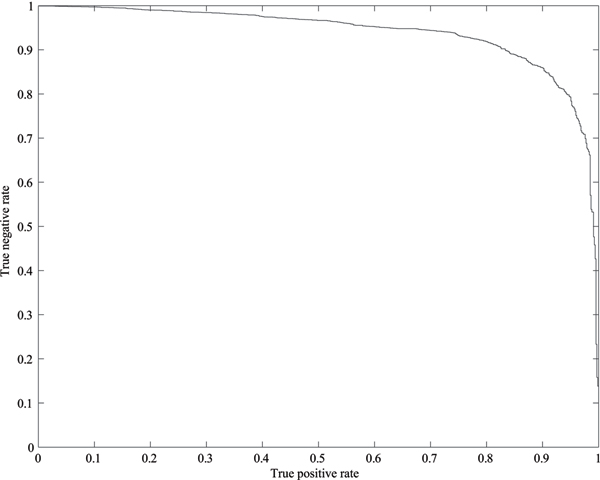
**ROC curve for the SVM classifier for multiply charged ISB spectra**. Even if only 2% loss of high quality multiply charged spectra is allowed, the proposed method can filter out about 70% of the poor quality ones.

**Figure 2:**
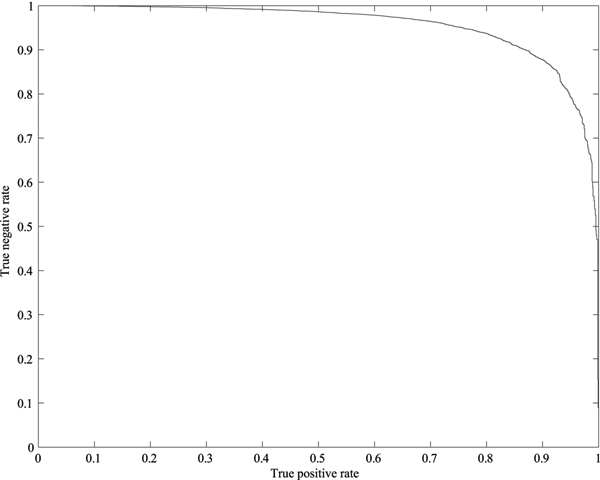
**ROC curve for SVM classifier for doubly charged TOV spectra**. Even if only 2% loss of high quality doubly charged spectra is allowed, the proposed mrthod can filter out over 65% of the poor quality ones.

In this study we repeated to train and test each SVM classifier on 20 randomly sampled datasets to investigate the performance of the proposed methods. The results are shown in Tables 4, 5, 6, 7. In these tables, 'Ave.' stands for the average and 'SD' for the standard deviation. For the doubly charged spectra of the ISB dataset, Table 4 shows that the proposed method can eliminate about 89% of the poor quality spectra while losing less than 6% of the high quality spectra at the best case. On average the SVM classifier can eliminate about 90% of the poor quality spectra while losing less than 8% of the high quality spectra. For the triply charged spectra of the ISB dataset, Table 5 shows that the proposed method can remove more than 87% of the poor quality spectra while losing about 4% of the high quality spectra at the best case. On average it can remove about 88% of the poor quality spectra while losing about 7% of the high quality spectra. Table 6 shows that the proposed method can remove over 87% of the poor quality multiply charged spectra (not distinguishing between doubly charged and triply charged ions) while losing less than 8% of the high quality ones by using the ISB dataset as the classification test at the best case. On average it can remove over 87% of the poor quality multiply charged spectra while losing about 9% of the high quality ones. For the TOV dataset, Table 7 shows that the developed SVM classifier can remove about 85% of the poor quality spectra while losing less than 5% of the high quality ones at the best case. On average about 84% of the poor quality spectra can be removed while losing less than 8% of the high quality spectra by using the TOV dataset as the classification test. In summary, the four SVM classifier developed in this study performs very well. In addition, comparing Table 6 with Tables 4 and 5, it indicates that the information about the charge state of the precursor ions can be used to improve the performance of the SVM classifiers. Figures 1 and 2 show the ROC curves for the SVM classifier for multiply charged ISB dataset and the SVM classifier for doubly charged TOV dataset, respectively. Even if only 2% loss of high quality spectra is allowed, the proposed method can filter out about 70% of poor quality multiply charged ISB spectra and 65% of poor quality doubly charged TOV spectra, respectively.

**Table 4:** The results in the ISB test data with the SVM classifier for doubly charged spectra

Times	FP	FN	TPR (%)	TNR (%)
1	1608	64	92.1	90.4

2	1631	69	91.5	90.3

3	1853	52	93.6	89.0

4	1879	46	**94.3**	**88.8**

5	1719	63	92.2	89.8

6	1887	55	93.2	88.8

7	1633	65	92.0	90.3

8	1667	74	90.9	90.1

9	1643	70	91.4	90.2

10	1660	80	90.2	90.1

11	2070	59	92.7	87.7

12	1723	58	92.9	89.8

13	1739	73	91.0	89.7

14	1813	74	90.9	89.2

15	1667	68	91.6	90.1

16	1921	57	93.0	88.6

17	1793	54	93.4	89.3

18	1756	77	90.5	89.6

19	1767	55	93.2	89.5

20	1653	73	91.0	90.2

Ave.	1754	64	92.1	89.6

SD	120.5	9.5	1.17	0.72

**Table 5:** The results in the ISB test data with the SVM classifier for triply charged spectra

Times	FP	FN	TPR (%)	TNR (%)
1	2120	24	91.2	87.7

2	2091	15	94.5	87.8

3	2195	12	**95.6**	**87.2**

4	2324	19	93.0	86.5

5	2029	26	90.5	88.2

6	1952	23	91.6	88.6

7	2163	12	95.6	87.4

8	2350	19	93.0	86.3

9	1967	20	92.7	88.5

10	1994	21	92.3	88.4

11	2071	17	93.7	87.9

12	1948	21	92.3	88.7

13	2163	26	90.5	87.4

14	1998	26	90.5	88.4

15	2162	23	91.6	87.4

16	2101	16	94.1	87.8

17	2005	21	92.3	88.3

18	2161	20	92.7	87.4

19	1930	28	89.7	88.7

20	2134	17	93.8	87.6

Ave.	2093	20	92.7	87.8

SD	118.3	4.6	1.67	0.69

**Table 6:** The results in the ISB test data with the SVM classifier for multiply charged spectra

Times	FP	FN	TPR (%)	TNR (%)
1	4412	121	90.8	87.3

2	4430	101	91.7	87.2

3	4663	118	90.3	86.5

4	4348	106	91.2	87.4

5	4337	122	89.9	87.5

6	4639	106	91.2	86.6

7	3944	121	90.0	88.6

8	4444	103	91.5	87.2

9	4684	109	91.0	86.5

10	4705	92	92.4	86.4

11	4296	109	91.0	87.6

12	4383	114	90.6	87.3

13	4342	121	90.0	87.5

14	4485	94	**92.2**	**87.1**

15	4197	114	90.6	87.9

16	4604	107	91.2	86.7

17	4499	110	90.9	87.0

18	4007	131	89.2	88.4

19	4009	138	88.6	88.4

20	4275	111	90.8	87.7

Ave.	4385	112	90.7	87.3

SD	223.7	11.4	0.94	0.65

**Table 7:** The results in the TOV test data with the SVM classifier for doubly charged spectra

Times	FP	FN	TPR (%)	TNR (%)
1	814	31	90.2	84.4

2	925	23	92.7	82.3

3	839	29	90.9	83.9

4	911	29	90.9	82.6

5	856	24	92.4	83.6

6	800	22	93.1	84.7

7	816	30	90.5	84.4

8	920	15	95.3	82.4

9	788	27	91.5	84.9

10	799	27	91.5	84.7

11	790	30	90.5	84.9

12	787	15	**95.3**	**84.9**

13	922	29	90.9	82.4

14	766	27	91.5	85.3

15	957	18	89.9	81.7

16	819	28	91.2	84.93

17	871	22	93.1	83.3

18	885	25	92.1	83.1

19	830	27	91.5	84.1

20	823	23	92.7	83.8

Ave.	846	25	92.1	83.8

SD	56.4	4.8	1.51	1.08

Table 8 gives the correct rates of the proposed method and some of the existing methods. From Table 8, it can be seen that the performances of the methods early proposed by Tabb *et al*. [8] and Purvine *et al*. [9] are not good and do not give the values of TPRs. While the TPR is fixed at 90% (i.e., the percentage of losing high quality spectra is fixed at 10%), the best results reported by the existing methods from [11] and [14] are that the TNR is about 83%. For the ISB dataset, the TNRs in [13] and [14] are obtained at 75% and 83%, respectively. However, for the multiply charged spectra of ISB dataset, on average the proposed method can remove more than 87% of the poor quality spectra while losing about 9% of the high quality ones. This illustrates that the proposed method outperforms these existing methods.

**Table 8:** Correct rates of the proposed method and some existing methods

	TPR (%)	TNR (%)
SVM2ISB	92.1	89.6
SVM3ISB	92.7	87.8
SVMMISB	90.7	87.3
SVM2TOV	92.1	83.8
Flikka *et al*. [11]	90.0	83.0
Wong *et al*. [14]	90.0	83.0
Bern *et al*. [10]	90.0	75.1
Salmi *et al*. [12]	90.0	75.0
Na *et al*. [13]	90.0	75.0
Purvine *et al*. [9]	-	55.0
Tabb *et al*. [8]	-	40.0

In addition, Wong *et al*. [14] did not report the performance of their proposed method for randomly sampled datasets. Bern *et al*. [10] just reported the best results from their methods. In [11] and [13], the 5-fold cross validation was employed to test their proposed approaches. However, they did not report the entire results across per test fold. In [12], the 10-fold cross-validation was applied to assess the performance of their proposed method. While the TPR is fixed about 90%, the TNR is varied between 43% and 75%, which means that their proposed method is highly sensitive to the variations of the training and test sets in the same dataset. However, the last row in each of Tables 4, 5, 6, 7 gives the standard deviation of our proposed methods over twenty randomly sampled datasets. All the standard deviations for TPR and TNR are very small (from 0.65%–1.67%). This indicates that the proposed method is insensitive to the variations of the training and test sets in the same dataset.

The SVM classifiers are trained with the results of SEQUEST which may also have false positive or false negative. To further illustrate the bias raised from the SEQUEST results, we have investigated the spectra in the false positive set and the false negative set, respectively. For this purpose, we randomly selected 100 false positive spectra (50 doubly charged and 50 triply charged) from the ISB dataset, which are classified as high quality by the proposed method, yet were unidentified by the SEQUEST search program. These 100 spectra were re-searched by on-line MASCOT [24] against the SwissProt database. The parent mass tolerance was set at ± 2 Da, and the fragment ion mass tolerance was set at ± 0.5 Da. The enzyme parameter was set as tryptic sequences, and the maximum of missed cleavage site was 1. We found that 14 doubly charged and 8 triply charged spectra had peptide-spectrum matching scores over the cut-off for peptides with significant homology. This indicates that 22% of false positives may be true positive, thus the TNR of our method should be higher. In addition, the 15 spectra out of these 22 spectra are interpreted as the same peptides by SEQUEST (below the cut-off score) and MASCOT (above the cut-off score).

**Figure 3:**
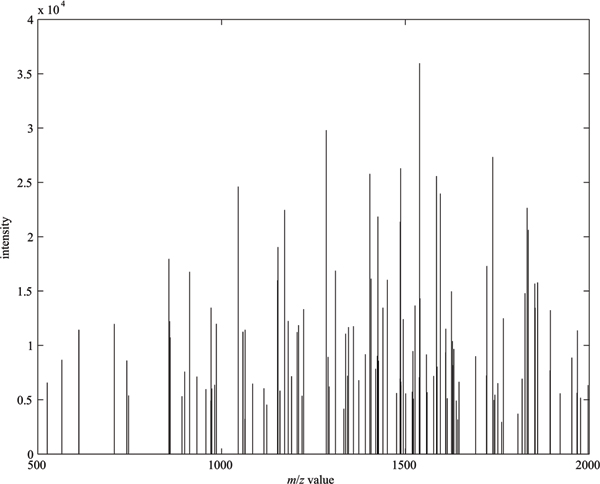
A false negative spectrum from the ISB dataset.

We also randomly selected 10 false negative spectra, which have high matching scores from SEQUEST method, yet are classified as poor quality by the proposed method. These 10 spectra were also re-searched by on-line MASCOT [24] against the SwissProt database. We found that the MASCOT ion scores of all these ten spectra were less than 15. This indicates that these 10 spectra may be true negative. To confirm this indication, manual verification by a mass spectrometry expert is required. Figure 3 shows one of these spectra, which was interpreted as doubly charged ion with peptide 'VAGTWYSLAMAASDI SLLDAQSAPLR' by SEQUEST with a high Xcorr score 3.4848. However, its MASCOT ion score is as low as 2. From Figure 3, it is obvious that this spectrum is poor quality because noisy and signal peaks are undistinguishable.

## Conclusion

In this paper, an SVM-based method is proposed for assessing the quality of tandem mass spectra from ion trap mass spectrometers. 16 spectral features are introduced to describe the quality of peptide mass spectra. Each spectrum is mapped into a 16-dimensional feature vector. The SVM is applied to construct the classifier in the feature space that distinguishes the high quality from the poor quality of peptide mass spectra. Four separate SVM classifiers are trained and tested on two different mass spectral datasets: ISB and TOV datasets. Computational experimental results have demonstrated the effectiveness of the proposed method and indicated that the proposed method outperforms the existing methods.

The significance of the proposed method is three-fold. First, the proposed method provides a reliable evaluation of the spectral quality. Therefore, the poor quality spectra can be filtered out before database search, which significantly reduces the computational time on spectral searching. Second, the proposed method can be employed to evaluate the database search results from one search engine while incorporating with different identification methods. For example, by both re-searching false negative spectra with MASCOT and manual verification, we can confirm that assignments of these spectra by SEQUEST are actually false. Third, the proposed method can be used to identify more significant peptide-spectrum assignments. For example, in this study by searching the false positive spectra (which are determined to be of high quality by the proposed method) with MASCOT, about 22% of these spectra are identified. Although all database searching assignments and our proposed method did not take the post-translationally modified amino acids except for sufloxidized Methionine in this study, our method still can be beneficial in identifying post-translationally modified peptide/proteins. Actually each protein has only a few modified amino acids and each peptide has much fewer modified amino acids. The values of our proposed features may be affected little by only a few modified amino acids. Because of the robustness of our methods, the spectra with modified amino acids are likely determined as high quality. Note that the number of high quality spectra determined by the proposed method is much less than the original spectral dataset. Therefore, it would save a significant amount of time to find post-translationally modified peptides/proteins by just searching those high quality spectra.

## Competing interests

The authors declare that they have no competing interests.

## Authors' contributions

AMZ developed the algorithm, designed and conducted all experimental work, and wrote the first draft. JRD participated in the software development, discussion and proofreading of the manuscript. FXW supervised and coordinated this project. FXW and GGP initiated this project and substantially revised the manuscript. All authors read and approved the manuscript.

## References

[B1] Eng JK, McCormack AL, Yates JR (1994). An approach to correlate tandem mass spectral data of peptides with amino acid sequences in a protein database. Journal of American Society for Mass Spectrometry.

[B2] Perkins DN, Pappin DJC, Creasy DM, Cottrell JS (1999). Probability-based protein identification by searching sequence database using mass spectrometry data. Electrophoresis.

[B3] Moore RE, Young MK, Lee TD (2002). Qscore: an algorithm for evaluating SEQUEST database search results. Journal of American Society for Mass Spectrometry.

[B4] Keller A, Nesvizhskii AI, Kolker E, Aebersold R (2002). Empirical statistical model to estimate the accuracy of peptide identifications made by MS/MS and database search. Analytical Chemistry.

[B5] Anderson DC, Li W, Payan DG, Noble WS (2003). A new algorithm for the evaluation of shotgun peptide sequencing in proteomics: support vector machine classification of peptide MS/MS spectra and SEQUEST scores. Journal of Proteome Research.

[B6] Razumovskaya J, Olman V, Xu D, Uberbacher EC, VerBerkmoes NC, Hettich RL, Xu Y (2004). A computational method for assessing peptide-identification reliability in tandem mass spectrometry analysis with SEQUEST. Proteomics.

[B7] Nesvizhskii AI, Roos FF, Grossmann J, Vogelzang M, Eddes JS, Gruissem W, Baginsky S, Aebersold R (2006). Dynamic spectrum quality assessment and iterative computational analysis of shotgun proteomic data. Molecular & Cellular Proteomics.

[B8] Tabb DL, Eng JK, Yates JR, James P (2001). Protein idebtification by SEQUEST. Proteome Research: Mass Spectrometry.

[B9] Purvine S, Kolker N, Kolker E (2004). Spectral quality assessment for high-throughput tandem mass spectrometry proteomics. OMICS.

[B10] Bern M, Goldberg D, McDonald WH, Yates JR (2004). Automatic quality assessment of peptide tandem mass spectra. Bioinformatics.

[B11] Flikka K, Martens L, Vandekerckhove J, Gevaert K, Eidhammer I (2006). Improving the reliability and throughput of mass spectrometry-based proteomics by spectrum quality filtering. Proteomics.

[B12] Salmi J, Moulder R, Filen JJ, Nevalainen OS, Nyman TA, Lahesmaa R, Aittokallio T (2006). Quality classification of tandem mass spectrometry data. Bioinformatics.

[B13] Na S, Paek E (2006). Quality assessment of tandem mass spectra based on cumulative intensity normalization. Journal of Proteome Research.

[B14] Wong JWH, Sullivan MJ, Cartwright HM, Cagney G (2007). msmsEval: tandem mass spectral quality assignment for high-throughput proteomics. BMC Bioinformatics.

[B15] Vincent L (1993). Morphological grayscale reconstruction in image analysis: application and efficient algorithm. IEEE Transaction on Image Processing.

[B16] Wu FX, Gagne P, Droit A, Poirier GG (2008). Quality assessment of peptide tandem mass spectra. BMC Bioinformatics.

[B17] Vapnik V (1998). Statistical Learning Theory.

[B18] Pontil M, Verri A (1997). Properties of support vector machines.

[B19] Platt J (1999). Fast training of support vector machines using sequential minimal optimization. Advances in Kernel Methods – Support Vector Learning.

[B20] Fawcett T (2006). An introduction to ROC analysis. Pattern Recognition Letters.

[B21] ftp://ftp.ncicfr.gov/pub/nonredun/protein.nrdb.Z.

[B22] Gagne JP, Gagne P, Hunter JM, Bonicalzi ME, Lemay JF, Kelly I, Le Page C, Provencher D, Mes-Masson AM, Droit A, Bourgais D, Poirier GG (2005). Proteome profiling of human epithelial ovarian cancer cell line TOV-112D. Molecular & Cellular Biochemistry.

[B23] Uniref100 Database. http://www.uniprot.org.

[B24] MASCOT. http://www.matrixscience.com.

